# ﻿SparkEC: speeding up alignment-based DNA error correction tools

**DOI:** 10.1186/s12859-022-05013-1

**Published:** 2022-11-07

**Authors:** Roberto R. Expósito, Marco Martínez-Sánchez, Juan Touriño

**Affiliations:** Universidade da Coruña, CITIC, Computer Architecture Group, Campus de Elviña, 15071 A Coruña, Spain

**Keywords:** Error correction, Big data, Distributed processing, Apache Spark

## Abstract

**Background:**

In recent years, huge improvements have been made in the context of sequencing genomic data under what is called Next Generation Sequencing (NGS). However, the DNA reads generated by current NGS platforms are not free of errors, which can affect the quality of downstream analysis. Although error correction can be performed as a preprocessing step to overcome this issue, it usually requires long computational times to analyze those large datasets generated nowadays through NGS. Therefore, new software capable of scaling out on a cluster of nodes with high performance is of great importance.

**Results:**

In this paper, we present SparkEC, a parallel tool capable of fixing those errors produced during the sequencing process. For this purpose, the algorithms proposed by the CloudEC tool, which is already proved to perform accurate corrections, have been analyzed and optimized to improve their performance by relying on the Apache Spark framework together with the introduction of other enhancements such as the usage of memory-efficient data structures and the avoidance of any input preprocessing. The experimental results have shown significant improvements in the computational times of SparkEC when compared to CloudEC for all the representative datasets and scenarios under evaluation, providing an average and maximum speedups of 4.9$\times$ and 11.9$\times$, respectively, over its counterpart.

**Conclusion:**

As error correction can take excessive computational time, SparkEC provides a scalable solution for correcting large datasets. Due to its distributed implementation, SparkEC speed can increase with respect to the number of nodes in a cluster. Furthermore, the software is freely available under GPLv3 license and is compatible with different operating systems (Linux, Windows and macOS).

**Supplementary Information:**

The online version contains supplementary material available at 10.1186/s12859-022-05013-1.

## Background

As the need to process large amounts of DNA sequences (the so-called reads) to conduct novel research keeps growing, new technologies grouped under Next Generation Sequencing (NGS) have arisen over the last decade to solve this requirement [1]. However, NGS platforms are not perfect and can introduce sequencing errors in the generated reads which can affect the quality of downstream analysis. Therefore, error correction is an important preprocessing step in many NGS pipelines (see Section 1 of Additional file 1 for more information about this topic).

Due to its importance, multiple correction algorithms have been proposed in the literature [2, 3]. However, most of the previous solutions usually lack either accuracy in correction, performance when processing large datasets, or the capability to scale out on a computing cluster. Among them, CloudEC [4] has been proved to perform precise corrections together with a scalable approach by relying on Big Data technologies, since its correction algorithms have been designed upon the MapReduce paradigm [5] using its most popular open-source implementation Apache Hadoop [6] (more details about Big Data and MapReduce are provided in Section 2 of Additional file 1). However, the usage of this tool comes at the cost of poor performance in terms of computational time when managing the huge amounts of data usually generated by NGS platforms. According to their own published results [7], the fastest experiment takes more than 18 h when correcting a dataset with 200 million reads on an 80-node computing cluster, showing a limited speedup of 5$\times$ (5 times faster execution time using 8 times the number of nodes). In order to overcome this problem, in this work we are introducing SparkEC as a new tool based on this previous approach that can tackle these scalability limitations without giving up either of its advantages in terms of correction accuracy.

The main contributions of this paper are:

- A new parallel tool based on Apache Spark [8] aimed at correcting errors in genomic reads that relies on accurate algorithms based on multiple sequence alignment strategies.
- A novel split-based processing strategy with a two-step k-mers distribution that allows correcting large NGS datasets much faster than previous tools.
- A simplified workflow for scientists and researchers by directly supporting standard unaligned formats without any need for input preprocessing.

### Related work

According to recent literature, current state-of-the-art correction approaches can be grouped into three main categories [9]: k-mer spectrum-based algorithms, suffix-tree based approaches, and strategies that rely on Multiple Sequence Alignment (MSA). The first category is based on grouping and counting subsequences of a fixed length *K* from the reads (i.e., the so-called k-mers). After this counting has been performed, k-mers are classified as solid or weak depending on their number of appearances. After that step, corrections on input reads are made to transform the weak k-mers into solid ones. The second category is an extension of the previous approach, where instead of keeping a hash table with all the different k-mers, the data structure to store them is based on a tree that keeps track of the different suffixes of the reads. This allows these algorithms to find low frequency strings composed by high frequency substrings, enabling them to easily spot the errors. Finally, MSA algorithms [10] are based on identifing groups of similar sequences and aligning them in order to construct a reference read that has more similarity with the original one. After this step, changes on input reads are made in order to near them to the original sequence.

MSA-based approaches typically allow for higher error correction precision but at the expense of greater computational complexity due to the multiple alignments. The CloudEC tool proposes two correctors that fit into this last category, and consequently our proposal also falls into this type of algorithms.

#### Big data and parallel correctors

Big Data technologies are increasingly being used to handle the processing of large genomic datasets in a scalable way, including aligners and assemblers, among others [11–16]. In the context of DNA error correction, multiple solutions have been proposed in recent years. If we delve into the literature, there exist representative works for each one of the three aforementioned correction strategies: (1) those that count the frequency of the different substrings or k-mers in order to spot misread bases (e.g., Reptile [17], BLESS2 [18], Musket and its Spark-based approach [19, 20]); (2) tools that generalize the previous strategy by using trees to analyze the suffixes of the strings (e.g., Pluribus [21], SHREC [22]); and (3) correctors that rely on MSA strategies to make multiple alignments of the input reads to apply the corrections among them [23, 24].

Delving into MSA-based parallel tools, the ALLPATHS-LG assembler [25] provides a built-in error corrector implemented using a set of tasks that are executed through a Makefile. Therefore, it easily allows to set up a multi-threaded execution by taking advantage of the support provided by Makefile for such goal. However, it is not possible to distribute the computation across a cluster of nodes with this approach. For this reason, CloudRS [26] has been proposed by taking the corrector of ALLPATHS-LG as baseline and implementing it upon Apache Hadoop. Thanks to this change, CloudRS is able to process the sequences using multiple worker nodes, effectively allowing it to handle larger datasets than ALLPATHS-LG in less time. Finally, CloudEC [7] is another Hadoop-based MSA corrector that was presented as an enhanced version of CloudRS. The major improvement of CloudEC over its counterpart was the introduction of the *spread corrector*, a new MSA-based algorithm which increases the reliability of the reads at the cost of reducing its performance, as this algorithm is much more computationally intensive than the one provided by CloudRS (i.e., the *pinch corrector*). Although providing more accurate correction algorithms represents a clear advance in the state of the art, their usage in large datasets is unaffordable in terms of computational time. This is the main challenge that our proposal tries to overcome.

## Implementation

As mentioned earlier, the correction algorithms provided by our tool SparkEC are the ones originally proposed by the MSA-based reference tool CloudEC (i.e., the *spread* and *pinch correctors*). In this work, we did not take any action to enhance those correction algorithms in terms of their accuracy, since they have been extensively evaluated in previous works [7, 26]. Instead, our objective is twofold: increasing their performance by reducing the execution time, and improving their usability by removing the need from the user to manually execute some tedious tasks. These enhancements will be presented and analyzed throughout this section.

### CloudEC architecture

**Fig. 1:**
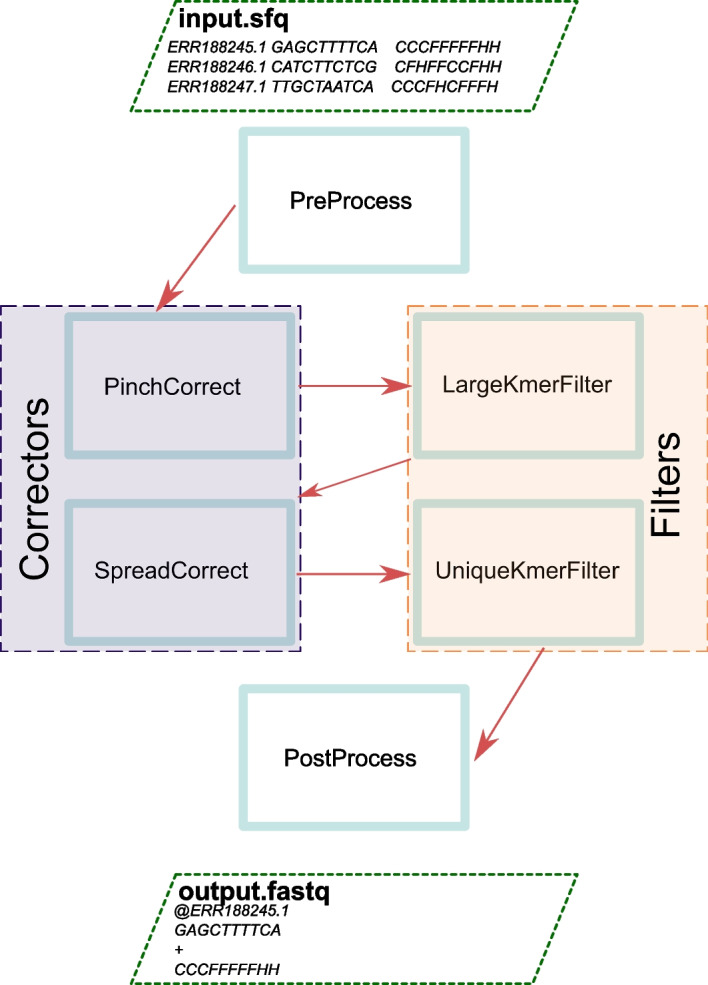
Phases of the CloudEC pipeline

Before getting into details about SparkEC, it is important to outline how CloudEC is structured at a high level. This tool makes use of the Pipe &Filter architectural pattern, which decomposes the whole job to be undertaken into multiple phases through which the data flow gets progressively transformed into the desired final result. More specifically, CloudEC consists of six phases: two correctors (PinchCorrect and SpreadCorrect), which are responsible of implementing the MSA-based algorithms themselves; two filters (LargeKmerFilter and UniqueKmerFilter), which speed up the execution of the correctors by tagging sequences that should not be processed; and two auxiliary phases (PreProcess and PostProcess), which handle both the input and output data flow of the tool, respectively. The overall pipeline defined with these six phases, which is depicted in Fig. 1, will be kept in our implementation except for minor changes during the preprocessing step aimed at supporting additional input formats, as will be later explained.

As previously mentioned, CloudEC is implemented upon the Hadoop framework (more details in Section 2.2 of Additional file 1). The procedure used to set up the pipeline described earlier with Hadoop is as follows: for each phase, a set of MapReduce jobs is dispatched one after another, chaining the output of a phase with the input of the next one. This tool defines a common internal data format among all the pipeline phases that is used for this intermediate communication between them. Furthermore, the dataset provided to CloudEC as input is expected to be stored in this custom format, requiring the user to preprocess the sequencing data on his/her own to convert the input reads before being able to run the pipeline. This additional preprocessing step means that CloudEC has seven effective phases, with the first one not being parallelizable. Moreover, this tool is unable to directly process standard sequence formats such as FastQ.

The execution of multiple MapReduce jobs to implement the pipeline has also some implications in terms of performance: firstly, a Shuffle & Sort task (see Section 2.1 of Additional file 1) has to be undertaken by the Hadoop data processing engine in most of the jobs, which degrades the overall performance of CloudEC due to the usage of network and secondary storage for temporary data (e.g., data shuffling); secondly, data processed by MapReduce jobs are typically stored in long-term storage, degrading even more the throughput of the application. With our first optimization detailed next we provide a solution to these two problems.

### Replacement of the data processing paradigm

The first step carried out was to migrate the underlying data processing engine from Hadoop to Spark. This transition implies the replacement of all the explicit MapReduce jobs executed by CloudEC to the specific paradigm defined by Spark, based on an implicit handling of these tasks via the usage of abstract data transformations.

To do so, the Resilient Distributed Datasets (RDDs) [27] defined by Spark were used. These structures allow the developer to store data that are defined as a set of elements in a distributed way across a cluster of nodes. Details about RDDs are provided in Section 2.3 of Additional file 1. Their interesting features, such as the support of in-memory computations in a fault-tolerant manner, are specially beneficial for the performance of our tool. In fact, they can either solve or, at least, reduce the impact of the two CloudEC problems mentioned earlier: (1) most of the operations performed over RDDs are lazily computed, enabling Spark to coalesce some of them and thus minimizing the Shuffle & Sort tasks that have to be undertaken; and (2) since the RDDs can be stored into main memory, the usage of secondary storage can be reduced, improving the overall performance. Moreover, the RDDs can be cached to prevent Spark from disposing them, being able to reuse the data previously generated. In SparkEC, this functionality is applied to the input reads, keeping always the most recent version of the dataset cached in memory. This configuration allows our tool to generate the k-mers that will be used by both correctors and filters and, after applying the corresponding algorithm, join those k-mers again with their original sequences.

Finally, Spark also allows developers to manage both the number of partitions and the partitioning strategy used for each RDD to determine how many pieces an RDD is decomposed into and the algorithm to assign the RDD elements to each partition, respectively. In our proposal, we have chosen to customize the default Spark behaviour when none of these values are provided, by keeping the number of partitions constant throughout all the execution. This way, we prevent the use of an extremely low number of partitions since the default behaviour of Spark is to set this value to the default parallelism level. Moreover, SparkEC relies on a hash-based partitioning strategy, which partitions the data based on the hashcode of the RDD elements. This strategy works well in our context and does not introduce the overhead of the range-based partitioning approach that is needed to guarantee that all partitions have the same size [28]. Furthermore, in order to achieve a homogeneous distribution with the hash-based partitioner, SparkEC defines the hashcode of the RDD elements in such a way that they get oddly distributed.

### Input preprocessing

Another important drawback of the CloudEC tool is the need to preprocess the input dataset to transform the FastQ sequences into an internal custom format named SimpleFastQ (SFQ). This step is specially heavy, since CloudEC does not provide any parallel approach that could take advantage of multiple nodes or threads. Only after executing this preprocessing step, the user can upload the transformed dataset to a distributed file system, such as the Hadoop Distributed File System (HDFS) [29], in order to be corrected in parallel by CloudEC.

To solve this issue, SparkEC relies on the the Hadoop Sequence Parser (HSP) [30]. HSP is a Hadoop-based library written in Java that allows developers to read both genetic and protein sequences stored in formats commonly used in the field (i.e., FastQ/FastA) from either local or distributed file systems such as HDFS. In our proposal, this library is introduced into the PreProcess phase (see Fig. 1), removing the requirement of preprocessing the input dataset and thus further optimizing performance. It is important to note that this optimization not only improves performance but also simplifies the overall pipeline that the users need to set up, enhancing the overall usability of the tool.

### Split-based system

By replacing Hadoop with Spark we can take advantage of its advanced features and extensions compared to the MapReduce model. However, simply replacing the underlying data processing engine would be a naive approach. A certain computing algorithm that works using the secondary storage might not perform adequately when constrained to use the main memory, as there is typically less memory than storage space available on disk. The default behaviour of Spark in such scenario, where it gets overwhelmed by the memory needs, is either to discard and recompute the RDD partitions as needed or instead to use local disks to store them, thus reducing the potential benefits of in-memory computations. Therefore, CloudEC is a clear example where a straightforward code migration from Hadoop to Spark may bring little to no performance advantage, since this tool provides very precise but memory-intensive correction algorithms that directly challenge the way Spark processes data in memory.

Therefore, a thorough redesign of CloudEC was mandatory in order to fully exploit Spark performance. To do so, SparkEC introduces a novel split-based processing system to keep the aggregate memory usage bounded during the computations. This optimization consists in preventing the alignment of all the sequences simultaneously. To achieve this behaviour, our tool starts by computing the total amount of memory available to Spark by multiplying the memory assigned to each Spark executor by the number of available executors (see Section 2.3.2 of Additional file 1 for an overview of Spark cluster deployment). Next, an estimation *M* of the memory required to process the input dataset is calculated as shown in Equation 1, with *L* being an estimate of the average length of DNA reads, *K* the length of the k-mers, *N* the number of total reads, and *C* a constant with a default value of 5.25 obtained as a result of an experimental tuning, but such value is configurable by the user (see Section 3.4 of Additional file 1).

1 $$\begin{aligned} M = ((L - K) * K) * N * C \end{aligned}$$

**Fig. 2:**
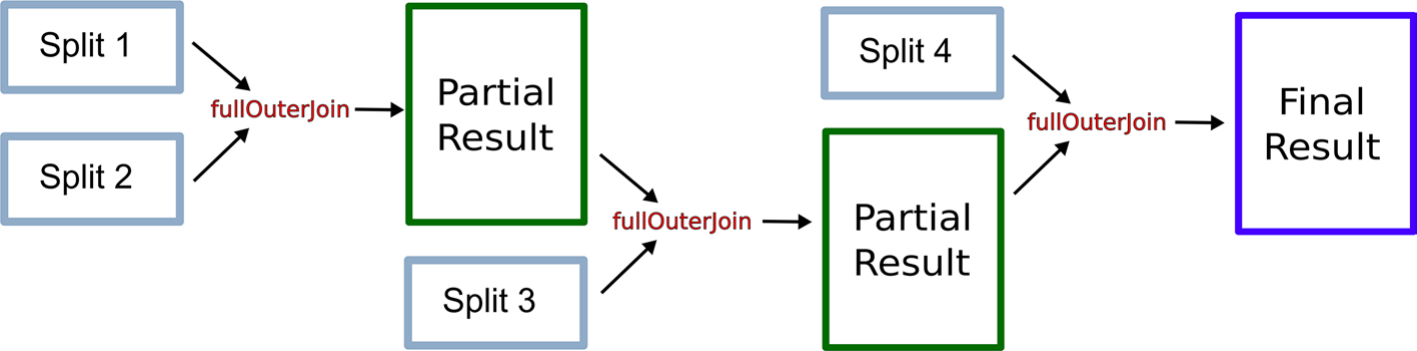
The split-based system over SpreadCorrect

Taking into account such memory estimation, a certain number of splits is recommended for each of the pipeline phases defined in SparkEC. Each phase will issue only the k-mers that are expected to be aligned in each split. After processing the different splits, the tool will aggregate the partial results that are generated, thus completing the execution of the whole phase. As an example, Fig. 2 depicts the overall execution workflow of the SpreadCorrect phase using the split-based system, showing the join-like Spark operations needed to aggregate partial results.

Finally, it is important to mention the limitations of our current implementation. On the one hand, the maximum sequence length (*L*) is limited to 32,767 base pairs, inherited from the CloudEC implementation. Longer sequences are omitted in SparkEC during processing, whereas CloudEC just fails at runtime. On the other hand, *L* is estimated in Equation 1 by taking a sample from the input dataset, so the memory estimation (*M*) may not be optimal for those datasets where there is a large variability in sequence length. Although the constant *C* can be set through configuration in order to tune the split-based system in those scenarios, it would be great to provide a heuristic to help determine a suitable value for such constant. However, the experimental results shown later, which include datasets containing fixed- and variable-length sequences, will demonstrate the effectiveness of the split-based system in its current form.

#### Two-step k-mers distribution

There is still a challenge to be considered when introducing the split-based system. Since Spark distributes the RDDs into different partitions in order to assign tasks to the available worker nodes to process them, it is necessary to prevent a potential collision between the partitioning algorithm used by Spark and the distribution of k-mers into splits performed by our tool. Such collision may arise due to the combination of the following three facts: (1) as explained before, the RDD elements are distributed among the partitions based on their hashcode; (2) the splits where a given data record belongs to are also assigned based on their hashcode; and (3) the algorithm that determines the partition where the RDD elements belong to using the hashcode is the same used to find out the split where they should be computed (i.e., the modulus between the hashcode and the number of either partitions or splits). Taking all these facts into account, in the event that the number of partitions and the number of splits have a common divisor, some workers would not perform any computation. For example, if both the number of partitions and the number of splits are divisible by 2, only the first half of workers would have workload assigned in the first split, and the second half only during the second split.

To overcome this issue, the split-based system distributes the k-mers in two steps: in the first one, all the k-mers are distributed into *P* groups based on their hashcode, being *P* greater than the number of splits (*S*) and co-prime with the number of partitions of the input RDD. In the second step, data are redistributed into the different splits by computing the modulus between its group number and *S*. This way, two different kinds of splits are created: the first $( P - S )$ splits will be assigned a given workload, and the remaining ones will have to handle only half of such workload. Therefore, the problem of having idle workers is solved, although at the cost of introducing a small workload imbalance between the splits.

The split-based system together with the two-step k-mers distribution has been experimentally proven to allow SparkEC to be significantly faster than CloudEC, even in scenarios where there was no enough memory to handle the datasets. This will be experimentally shown in the Results and Discussion section, where SparkEC takes advantage of this optimization specifically in those scenarios with heavy memory constraints (i.e., those using a low number of nodes).

### Memory-efficient data structures

Aligned with the previous optimization, we have also modified the representation of the internal data structures used by CloudEC in order to make them more efficient in terms of memory usage.

When using the original approach proposed by CloudEC, most of the communications made between the pipeline phases are undertaken by encoding the data into plain text (typically, transforming each of the fields to text and separating them with a tab character). In SparkEC, this data encoding has been replaced by using ad hoc classes that have specific fields defined with the minimum memory usage required. As an example, whereas CloudEC would use the textual representation of the identifier for each DNA read, SparkEC relies on a single long value to store it. Although it may not seem to have a huge impact, it is worth noting that the underlying correction algorithms are based on applying multiple alignments to the data. So, the subsequences that are being aligned have to keep track of the read where they were found, which means that there is a huge number of read references stored in memory while this step is being executed.

Moreover, whenever Spark needs to perform a Shuffle (i.e., a redistribution of the data across the workers), it first needs to serialize the data, which is a costly operation in terms of CPU and disk usage. To improve data shuffling, SparkEC has been developed to take advantage of the Kryo serialization library [31], which has been benchmarked against the default Java serializers used by CloudEC, proving to have better performance and being able to serialize data faster and in a more compressed way [32]. This improvement, together with the aforementioned change in the representation of the data structures, is specially relevant in SparkEC since even though Kryo may also be used with CloudEC, the memory-bound approach taken by the Spark processing paradigm obtains a higher benefit from memory optimizations.

#### Optimized encoding of DNA reads

Additional classes have also been introduced in SparkEC to optimize the encoding of the most used data structures, such as the base sequences or the reads. In this context, by read we refer to a tuple containing the bases, their qualities, an identifier, and additional fields to allow the auxiliary tagging of the read throughout the pipeline phases.

To encode the reads, a Node class is proposed as a partial replacement of the Utils class found in CloudEC. Unlike Utils, this new class, shown in Figure S4 of Additional file 1, contains explicit fields to encode each one of the attributes of the reads, rather than storing them in a Java HashMap that introduces memory overhead. To encode the bases and qualities of the sequences, a more complex approach is taken. Whereas CloudEC encodes the bases as text, we offer a generic interface called IDNASequence with two different implementations: EagerDNASequence and LazyDNASequence (see Figure S5 of Additional file 1). The first one stores the bases using an array of bytes and whenever a transformation is applied to the sequence, all the bases get recomputed. The second one applies a shared-memory, lazy-based approach to the bases by not executing the computations requested over them until the bases of the sequence are queried. This can be specially relevant, since the correction algorithms usually generate a large number of k-mers for each read, so being able to store all the k-mers and the read where they were generated from in the same location can save memory. However, our experimental results did not show a clear performance enhancement by using the shared-memory approach, and so EagerDNASequence is the default implementation used by SparkEC. This may be caused by the overhead introduced in the sequences to allow memory sharing and the need to keep the data distributed across the workers, forcing LazyDNASequence to replicate the entire reads and not only the k-mers.

## Results and discussion

The experimental evaluation of SparkEC has been carried out comparatively with CloudEC on a high-performance computing cluster, both in terms of execution time and scalability. All the experiments have been conducted using the Big Data Evaluator (BDEv) tool [33], which focuses on the benchmarking of Big Data processing frameworks and the applications and workloads developed on top of them. BDEv has been configured to use the YARN scheduler [34] provided with Hadoop to manage the computational resources of the cluster nodes. Experiments using 5, 9 and 13 nodes have been executed to analyze the scalability of both tools, where each cluster size *n* can be understood as one master and $n-1$ worker nodes. The main hardware characteristics shared by all the cluster nodes are summarized in Table 1.

**Table 1:** Hardware characteristics of the cluster nodes

CPU model	2 x Intel Xeon E5-2660 Sandy Bridge EP
CPU clock frequency	2.20 GHz
Turbo clock frequency	3 GHz
Cores per CPU	8
Threads per core	2
L1/L2/L3 cache	32 KB/256 KB/20 MB
RAM memory	64 GB DDR3 1600 MHz
Storage	HDD 1 TB SATA3 7.2K rpm
Network interfaces	InfiniBand FDR & Gigabit Ethernet

### Datasets and software configuration

As shown in Table 2, six publicly available real datasets have been evaluated, named after their accession numbers in the Sequence Read Archive (SRA) [35] at the National Center for Biotechnology Information (NCBI) [36]. These input datasets provide a sufficiently representative sample since they have been obtained from different sequencing platforms (see third column in the table), varying both the number of total sequences from 5 to 26 million (see fourth column) and their length from 100 to several thousands of base pairs in the case of D5 and D6 (the fifth column shows their average read length and the last one the total number of bases).

**Table 2:** Public datasets used in the experimental evaluation

Tag	Accession number	Instrument model	#Reads	Length	#Bases
D1	SRR352384	Illumina Genome Analyzer II	26.0 M	152 bp	4.0 G
D2	SRR022866	Illumina Genome Analyzer II	12.8 M	152 bp	1.9 G
D3	SRR034509	Illumina Genome Analyzer II	10.3 M	202 bp	2.1 G
D4	SRR4291508	Illumina HiSeq 2000	25.2 M	100 bp	2.5 G
D5	SRR21018951	Oxford Nanopore MinION	6.6 M	285 bp^a^	1.9 G
D6	SRR2063079	PacBio RS II SMRT	5.1 M	361 bp^a^	1.8 G

^a^Average read length

Regarding the configuration of both tools, scenarios with two different values for *K* ($K=24$ and $K=55$) have been included since those are the most widely used values according to similar studies in the literature. This parameter determines the length of the k-mers that are used to make the alignments, and has direct implications in terms of performance: the higher the value for this parameter, the lower the number of k-mers generated, and thus less computation is done. Regarding software configuration, Table 3 shows the specific versions that have been used in all the experiments. The only specific setting of Spark that was modified for the SparkEC executions was the configuration of the Kryo serializer. At the same time, there was a fine tuning of the HDFS configuration used by both tools for best performance: the replication factor (i.e., the number of replicas to store each block) was set to 2, whereas the block size was set to 64 MB.

**Table 3:** Software configuration used in the experiments

OS	CentOS 7 (v7.7.1908)
JVM	OpenJDK 1.8.0_242
Hadoop	2.9.2
Spark	2.3.4
HDFS block size	64 MB
HDFS replication factor	2

Finally, all the results shown in this section represent the arithmetic average of a minimum of 5 measurements for each experiment. The observed standard deviations were not significant since all the experiments were run with the cluster nodes in a dedicated manner (i.e., the hardware was never shared by other users’ jobs running on the cluster), which makes the average value a suitable performance metric for this work.

### Analysis of the results

**Fig. 3:**
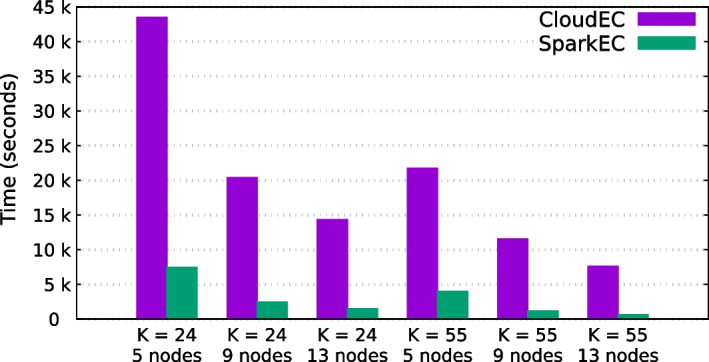
Runtimes for CloudEC and SparkEC when correcting D4

**Fig. 4:**
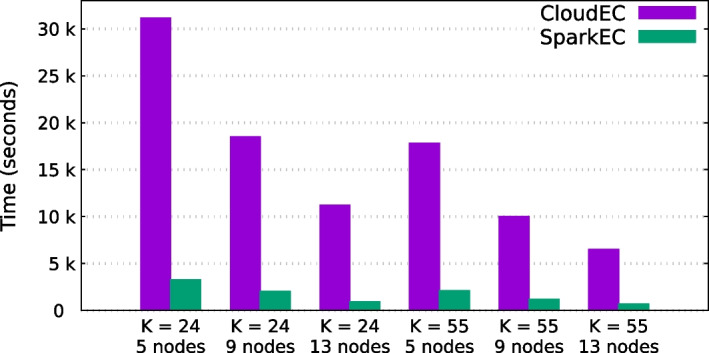
Runtimes for CloudEC and SparkEC when correcting D6

Table 4 presents the experimental results of the SparkEC and CloudEC tools for each dataset and k-mer length when using 5, 9 and 13 cluster nodes. Overall, these results evidence the significant performance gains that SparkEC provides over its Hadoop-based counterpart for all the scenarios under evaluation, achieving an average speedup of 4.9$\times$. On the one hand, this average speedup is around 4.3$\times$ for Illumina datasets (D1–D4), which contain short fixed-length reads (100–200 bp), reaching a maximum value of 11.8$\times$ when correcting the D4 dataset on 13 nodes using $K=55$ (see Fig. 3). This means that SparkEC can reduce correction times for this dataset from more than 2 h when using CloudEC to just 10 min. On the other hand, the results for long-read datasets (D5–D6), which contain variable-length reads, follow a similar trend. In this case, the average speedup is even higher (6.1$\times$), which validates the implementation of our split-based system when there is some variability in the length of the input reads. The maximum speedup is similar to that mentioned previously (11.9$\times$), obtained when correcting the D6 dataset on 13 nodes using $K=24$ (see Fig. 4). It is important to remark that all the results shown for CloudEC do not include the time needed to preprocess the input datasets in order to transform them into the custom format required by this tool. Therefore, if those times were to be added to the CloudEC runtime, the benefits of using SparkEC would be even greater. For illustrative purposes, the additional time needed to preprocess the D1 dataset is around 2 min, a fact that should be taken into account only when using CloudEC, whereas SparkEC avoids such preprocessing as previously explained, thus providing an overall faster and simplified workflow for end users.

**Table 4:** Runtimes (in seconds) and corresponding speedups of SparkEC over CloudEC for all datasets and k-mer values

Dataset	*K*	#Nodes	CloudEC	SparkEC	Speedup
D1	24	5	29,862	11,951	2.5
		9	13,697	4429	3.1
		13	9150	2731	3.4
	55	5	21,909	9693	2.3
		9	10,135	2792	3.6
		13	6216	1785	3.5
D2	24	5	13,307	5289	2.5
		9	5351	1659	3.2
		13	3309	971	3.4
	55	5	8688	4035	2.2
		9	3889	1250	3.1
		13	2594	700	3.7
D3	24	5	11,609	4865	2.4
		9	4831	1885	2.6
		13	3167	1113	2.8
	55	5	8502	4892	1.7
		9	3679	1348	2.7
		13	2616	756	3.5
D4	24	5	43,506	7473	5.8
		9	20,383	2484	8.2
		13	14,334	1511	9.5
	55	5	21,723	3987	5.4
		9	11,543	1146	10.1
		13	7617	648	11.8
D5	24	5	26,959	8607	3.1
		9	12,611	6486	1.9
		13	7729	3927	2.0
	55	5	14,374	3698	3.9
		9	5827	2233	2.6
		13	3577	1437	2.5
D6	24	5	31,146	3286	9.5
		9	18,511	2047	9.0
		13	11,232	942	11.9
	55	5	17,806	2105	8.5
		9	10,021	1180	8.5
		13	6514	687	9.5

**Fig. 5:**
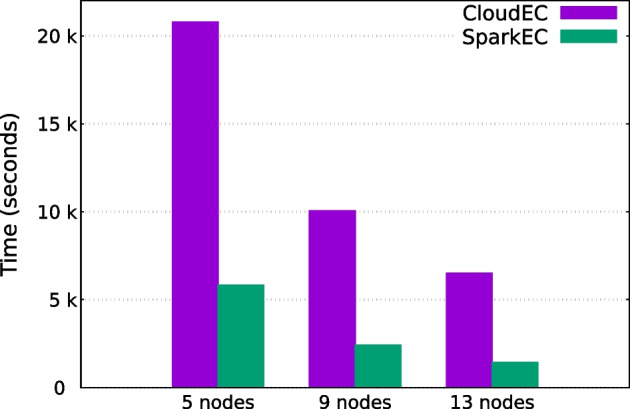
Runtimes for all datasets and *K* values grouped by number of nodes

It is also interesting to analyze the horizontal scalability provided by both tools, a feature which allows to further reduce the execution times by increasing the number of workers. The scalability results are shown in Fig. 5, where the runtimes obtained for each dataset and k-mer length are grouped together using an arithmetic average. As can be observed, the scalability provided by CloudEC is not only kept by our tool, but even enhanced. Whereas CloudEC is able to obtain an average runtime reduction of 69% when increasing the number of nodes from 5 to 13, SparkEC further increases such runtime reduction to 75%. Thus, the average speedups obtained by our tool over CloudEC range from a speedup of 4.0x when using 5 nodes up to 5.6x when using 13, representing a 40% boost.

**Fig. 6:**
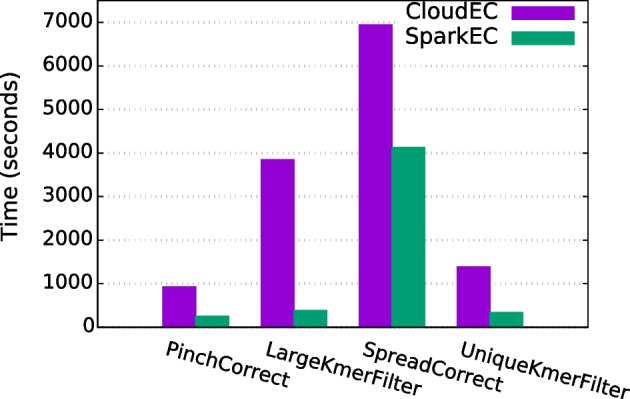
Runtime breakdown by phase when correcting D2 on 5 nodes

#### Runtime breakdown

**Fig. 7:**
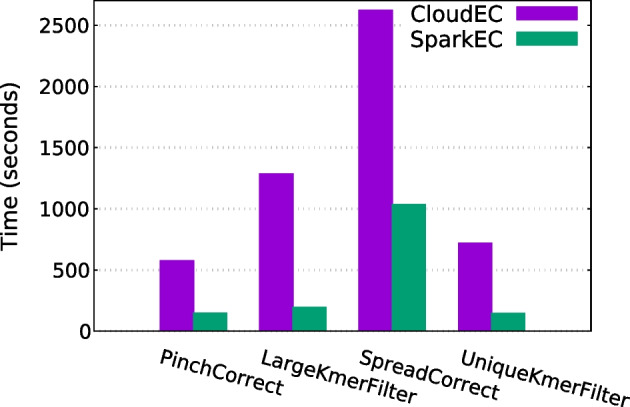
Runtime breakdown by phase when correcting D2 on 9 nodes

**Fig. 8:**
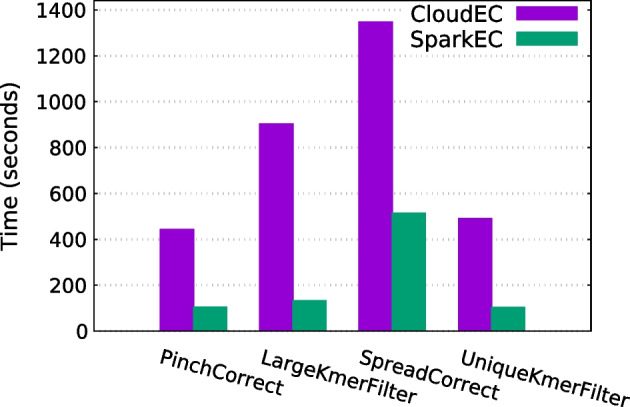
Runtime breakdown by phase when correcting D2 on 13 nodes

Finally, a further analysis of each individual pipeline phase has been undertaken to evaluate the results in more detail, excluding the preprocessing and postprocessing steps as their impact on the total execution time is relatively low. For this assessment, the scenario correcting the D2 dataset with $K=24$ has been selected, comparing the runtimes for each phase when using 5, 9 and 13 nodes. The obtained results are shown in Figs. 6, 7 and 8, respectively. As can be seen, SparkEC clearly outperforms CloudEC in all the phases regardless the number of nodes. Furthermore, the performance improvements provided by SparkEC are higher in those phases which are mostly compute-bound, as it is the case of PinchCorrect, LargeKmerFilter and UniqueKmerFilter. The opposite occurs in SpreadCorrect, since it is mostly an I/O-bound phase due to the large amount of data being shuffled by both Spark and Hadoop, although SparkEC keeps being able to provide significant speedups over CloudEC (up to 2.6x, see Fig. 8). This analysis also ensures that SparkEC would keep surpassing CloudEC even in those executions where some of the phases could be disabled, an advanced setting of both tools that can be configured by the user.

## Conclusion

As the amount of genomic data generated by NGS technologies continues to grow, so does the need for more efficient ways of storing and processing them. To improve the quality of downstream analyses, there exist many tools for error correction of such sequencing data, where MSA-based algorithms represent a computational challenge when correcting large datasets.

Under the light of the results presented in this work, it is clear that our proposal represents an advance in the state of the art of MSA-based correction algorithms. SparkEC significantly outperforms its Hadoop-based counterpart in all the experiments, providing maximum speedups of around 12$\times$ both for short- and long-read datasets. Our tool has also shown the ability to horizontally scale better than CloudEC and to perform well in resource-constrained scenarios and when correcting long-read datasets with variable-length sequences. Furthermore, SparkEC not only reduces the correction times to speed up subsequent biological research, but also simplifies its usage avoiding any preprocessing of the input reads. These characteristics will definitely contribute to lower the hardware requirements needed to apply MSA-based correctors after the DNA sequencing process, thus broadening the target scientists that can make use of these solutions. The result of this work is freely available under the permissive GPLv3 license and can be downloaded from the GitHub repository: https://github.com/UDC-GAC/SparkEC. Section 3 of Additional file 1 includes a user’s guide that provides detailed instructions about downloading, executing and configuring SparkEC.

As future work, there are some minor enhancements to further boost the performance of the tool. In the short term, the memory representation of some data structures may be optimized, and the partitioning strategy could be improved to better handle scenarios with a high number of splits. In the long term, where more memory could be available, the split-based system may be revamped to process all the data simultaneously and thus further reduce the execution time.

## Availability and requirements

Project name: SparkEC.

Project home page: https://github.com/UDC-GAC/SparkEC.

Operating system(s): Platform independent.

Programming language: Java.

Other requirements: JRE 1.8 or higher, Apache Spark 2.0 or higher, Apache Hadoop 2.8 or higher (needed for HDFS).

License: GNU GPLv3.

Any restrictions to use by non-academics: None.

## Supplementary Information

**Additional file 1**. Document including background information, additional figures related to the main text and a detailed user’s guide for SparkEC.

## Data Availability

The software, documentation and source code of SparkEC are publicly available at the GitHub repository: https://github.com/UDC-GAC/SparkEC. The real datasets analyzed during this study are also publicly available at the NCBI SRA repository (https://www.ncbi.nlm.nih.gov/sra) using the accession numbers: SRR352384, SRR022866, SRR034509, SRR4291508, SRR21018951 and SRR2063079.

## Abbreviations

NGS: Next generation sequencing
MSA: Multiple sequence alignment
RDD: Resilient distributed dataset
SFQ: SimpleFastQ
HDFS: Hadoop distributed file system
HSP: Hadoop sequence parser
BDEv: Big data evaluator
SRA: Sequence read archive
NCBI: National Center for Biotechnology Information
