# Supplementary material for: ﻿SparkEC: speeding up alignment-based DNA error correction tools
Source: BMC Bioinformatics. 2022 Nov 7;23:464. doi: 10.1186/s12859-022-05013-1 (PMC9639292; doi:10.1186/s12859-022-05013-1)
Supplement: Supplementary file 1 — Additional file 1. Document including background information, additional figures related to the main text and a detailed user’s guide for SparkEC. [file 12859_2022_5013_MOESM1_ESM.pdf]

SparkEC: speeding up alignment-based DNA error correction tools

Supplementary Material

Roberto R. Expósito, Marco Martínez-Sánchez, and Juan Touriño

1 NGS technologies and error correction

Nowadays, Next-Generation Sequencing (NGS) [1] is considered as an extremely useful technology for the prevention, diagnosis and treatment of a wide spectrum of diseases [2]. Several generations of sequencers have been introduced over the years to tackle the problem of generating DNA sequences from biological samples. With each generation, the number of reads that can be generated in a single run is increased, whereas the cost of the sequencing process is significantly reduced [3, 4]. Moreover, multiple bioinformatics tools have been developed to help in the task of assembling an entire genome from those reads. Some of them try to solve the entire problem at once [5], whereas others solve parts of a bigger pipeline that would allow to obtain a complete solution. A typical step of such pipelines is the alignment of the sequenced reads to a reference genome [6].

However, even though many improvements have been made in the latest NGS technologies, there are still some challenges to be addressed: firstly, none of the current sequencers is capable of generating DNA sequences that are free of errors, as stated in the literature [1, 7], which makes it necessary to maintain the concern of detecting and correcting those errors when working with NGS datasets; secondly, many modern sequencers are typically able to generate only short sequence fragments or reads, since the error rates rapidly increase with the length of such fragments. Therefore, another common pipeline task is the correction of errors introduced during the sequencing process. The underlying idea is that the prior usage of correction tools on raw NGS datasets provides assemblers with a cleaner input and subsequently leads to improved results.

Error correction has been addressed by multiple previous studies, which usually classify errors into three different types: 1) addition errors, where a new base that did not exist in the original sequence gets added to the read; 2) elimination errors, where an existing base is skipped from the read; and 3) substitution errors, which cause a base to be misread by another one (sometimes, even the presence of a base can be detected although the exact base cannot get determined). Regarding correction algorithms, existing approaches to detect and correct the aforementioned errors can be grouped into three main categories according to the literature [8]: k-mer spectrum-based algorithms, suffix-tree based approaches, and strategies that rely on Multiple Sequence Alignment (MSA) methods [9].

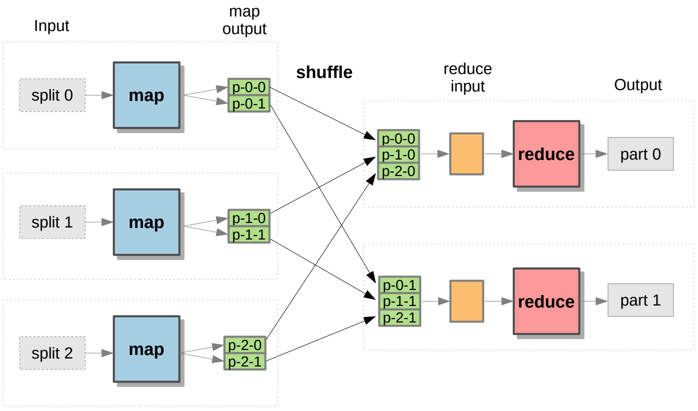

Figure S1. Overall workflow of the MapReduce paradigm

2 Big Data technologies

Scientists and researchers are currently facing new challenges when storing and analyzing massive datasets. The characteristics of Big Data require powerful and novel parallel approaches to extract meaningful information from such large datasets in a scalable manner by efficiently exploiting the computational resources of distributed-memory systems such as clusters and cloud platforms. The MapReduce programming paradigm [10] and its associated open-source implementation, the Apache Hadoop project [11], were the cornerstone of Big Data processing over the last decade.

2.1 The MapReduce paradigm

MapReduce is a parallel programming model and an associated implementation proposed by Google [10] for the storage and processing of large datasets over a cluster of commodity machines. This model allows transparent parallelization by relying on two user-defined functions: *Map* and *Reduce*. MapReduce adopts a data-parallel approach that first partitions the input dataset into multiple splits or chunks, each one containing many records in a <key,value> pair format, and then processes those splits in parallel by running multiple instances of the *Map* and *Reduce* functions (the map and reduce tasks). The user-defined *Map* function is first applied to transform the input <key,value> pairs into other intermediate ones (see Figure S1). After all map tasks have been completed, the intermediate pairs are sorted and grouped together according to their keys. Next, a shuffle phase is performed to transfer the intermediate pairs across the network so that all the values with the same key are sent to the same reduce task, which merges them into a single list to form the input of the *Reduce* function. Finally, the reduce tasks produce the final output also in the form of <key,value> pairs by applying the user-defined *Reduce* function.

2.2 Apache Hadoop

Apache Hadoop [11] is the most popular open-source MapReduce implementation derived from Google's proprietary one. Basically, Hadoop consists of three layers: (1) the Hadoop MapReduce engine as data processing layer; (2) the Hadoop Distributed File System (HDFS) [12] as storage layer; and (3) Yet Another Resource Negotiator (YARN) [13] as resource management layer.

On the one hand, HDFS is a block-oriented file system implemented in Java that mimics the Google File System (GFS) [14], being specifically designed to provide high bandwidth by distributing and replicating data across a cluster of commodity machines. This file system has built-in fault tolerance by using a data block replication scheme, and the number of times that each block is replicated over the cluster is defined as the replication factor. On the other hand, YARN manages the lifecycle of a distributed application by keeping track of the resources available on a computing cluster and allocating them for the execution of application tasks modeled after one of the supported computing paradigms such as MapReduce.

During the last decade, Hadoop and its ecosystem have become the most significant platform for Big Data processing. This framework generally shows good performance and scalability when executing embarrassingly parallel applications that require a single MapReduce job, provided that the volume of intermediate data between the map and reduce phases is not huge. However, one important limitation is its inefficiency for reusing intermediate results among several computations or MapReduce jobs, as Hadoop is a disk-based data processing engine. As a consequence, Hadoop MapReduce has been largely superseded by more advanced frameworks such as Apache Spark.

2.3 Apache Spark

Apache Spark [15] is an open-source, general-purpose Big Data framework. Spark overcomes the disadvantages of MapReduce by providing a richer programming API to allow for more flexible data-parallel operations, by reducing disk-based processing through in-memory computations, and by supporting streaming and interactive data processing, while also taking advantage of HDFS features.

2.3.1 Resilient Distributed Datasets (RDDs)

The fundamental data structure in Spark is based on the Resilient Distributed Dataset (RDD) [16], which provides an immutable, distributed collection of data elements partitioned across a cluster that can be operated in parallel and cached in memory to be reused in multiple MapReduce-like operations. RDDs can be created in different ways, for instance by loading an external dataset from supported file systems such as HDFS. Once created, an RDD can be manipulated using a rich set of data-parallel operations that can be classified into two types: transformations and actions. On the one hand, transformations (e.g., *map*, *filter*) are operations that create a new RDD from an existing one. For instance, the

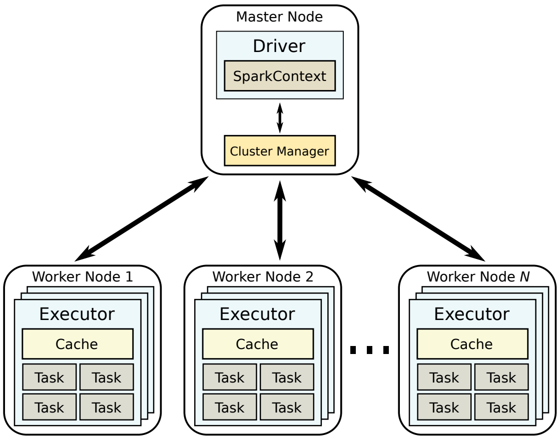

Figure S2. Overview of the Spark architecture and cluster deployment

map transformation processes each RDD element through a user-defined function and returns a new RDD representing the results. It is important to note that transformations are lazily evaluated, so they do not compute anything until an action that requires the result from them is triggered. On the other hand, actions are operations that return non-RDD values, converting the laziness of transformations into actual computation. They can be used either to return a result (e.g., *reduce*, *collect*), or to store the content of an RDD in external storage after running a certain computation (e.g., *saveAsTextFile*).

Regarding the internal implementation of RDDs, Spark keeps track of all the operations that have been performed over them, so that in the event of an error or data loss, it is possible to reconstruct the data by re-running all the operations again. By using this strategy, Spark is able to store the RDDs in a fault-tolerant way into the main memory of the cluster nodes, while also providing performance gains over Hadoop.

2.3.2 Spark cluster deployment

At a high level, Spark uses a master/worker architecture as depicted in Figure S2. The *Driver* program, which usually runs on the master node, executes the main function of the Spark application. For each application, the *Driver* first creates a *SparkContext* that acts as the central coordinator and then defines the RDDs and parallel operations to be carried out over them. Each worker node runs one or more *Executor* processes that are in charge of storing RDDs and effectively performing the computations. Tasks are the smallest computational units that can be run in parallel over an RDD by an *Executor*, and are scheduled on a per-core basis (i.e., one task per core). The *SparkContext* entity is responsible for creating, scheduling and sending individual tasks to be executed on the *Executors*, allocating the required computational resources through a cluster manager (e.g., Standalone, YARN).

3 SparkEC user's guide

SparkEC is a parallel tool that allows to correct DNA sequencing errors on NGS datasets, currently supporting the FastQ format. This project is based on the CloudEC tool [17], where its underlying MSA algorithms keep being the same in SparkEC, so their correction accuracy is ensured, but the code architecture has been completely refactored and the Hadoop framework replaced by Spark. Other novel optimizations provided by SparkEC include a split-based system using a two-step k-mers distribution, the avoidance of preprocessing of the input datasets and the use of more memory-efficient data structures.

3.1 Prerequisites

SparkEC is implemented in pure Java to maximize cross-platform compatibility. So, it runs on both Windows and UNIX-like operating systems (e.g., GNU/Linux, macOS), and it should run on any platform with a supported version of Java (x86_64 and ARM64).

The software prerequisites to run SparkEC are the following:

1. Apache Spark framework version 2.0 (or above). All you need is to have the `spark-submit` command available on your system PATH. To run SparkEC on a cluster, see Spark's cluster mode overview [18].
2. Java Runtime Environment (JRE) version 1.8 (or above) compatible with Spark. All you need is to have the `java` command available on your system PATH, or the JAVA_HOME environment variable pointing to your JRE installation.

To take advantage of HDFS to store and process the input datasets on a distributed manner, you also need Apache Hadoop version 2.8 (or above).

3.2 Download

To obtain SparkEC, you can download it from the GitHub repository[1] and unzip the tarball distribution (e.g., SparkEC-1.1.tar.gz). On UNIX-like operating systems, just execute the instruction below:

```
tar xzf SparkEC-1.1.tar.gz
```

Alternatively, you can clone the repository on your system by executing the following command:

```
git clone https://github.com/UDC-GAC/SparkEC.git
```

Note that the executable JAR file to run SparkEC (*SparkEC.jar*) is available at the *target* directory.

[1] `https://github.com/UDC-GAC/SparkEC/tags`

3.3 Execution

SparkEC must be executed by using the `spark-submit` command provided by Apache Spark, which launches the Spark jobs on the cluster. As mentioned before, you need to have the `spark-submit` command available on your PATH.

The general syntax to execute SparkEC is as follows:

```
spark-submit [spark_args] target/SparkEC.jar -in <input> -out
     <output> [-config <config_file>]
```

The meaning of the command-line arguments is:

- `spark_args`. Optional. These arguments are not interpreted by SparkEC, but they are forwarded to the Spark runtime[2]. For instance, you can use the `--master` option to set the master URL, depending on the cluster manager and execution mode (e.g., local[*], yarn, spark://host:port).
- `in`. Compulsory. String with the path to the input sequence file in FastQ format. It may be an HDFS or a local file system path.
- `out`. Compulsory. String with the path to the output directory used by SparkEC. It may be an HDFS or a local file system path.
- `config`. Optional. String with the path to the configuration file used by SparkEC. It must be a local file system path.

3.3.1 Local mode execution

To execute SparkEC locally, the `--master` option must be set to `local[N]`, being `N` the number of threads to be used. In this mode, the entire processing is done on a single server, so you still benefit from parallelization across all the cores in your computer, but not across several computers. When using `local[*]`, Spark uses as many threads as logical cores on your computer.

As an example, the following command corrects the dataset *sample.fastq* using the Spark local mode and 8 threads:

```
spark-submit --master local[8] target/SparkEC.jar -in sample.
    fastq -out sample-output
```

The following command shows a similar example but using as many threads as logical cores available on the server where this command is executed, specifying the path to a specific configuration file to be used:

```
spark-submit --master local[*] target/SparkEC.jar -in sample.
    fastq -out sample-output -config conf/config.properties
```

[2] `https://spark.apache.org/docs/latest/submitting-applications.html#launching-applications-with-spark-submit`

3.3.2 Cluster mode execution

To execute SparkEC on a cluster, the `--master` option must be set according to the cluster manager. Currently, Spark supports four cluster managers[3]: Standalone, Mesos, YARN and Kubernetes.

For the Spark standalone cluster mode[4], the `--master` option must be set to spark://host:port, where you must specify the hostname or IP address of the cluster master server and the port which is configured to be used (7077 by default).

As an example, the following command corrects the dataset *sample.fastq* on a Spark standalone cluster, where the master server IP is 207.184.161.138, using the client deploy mode:

```
spark-submit --master spark://207.184.161.138:7077 --deploy-
    mode client target/SparkEC.jar -in sample.fastq -out
    sample-output
```

When using YARN as cluster manager[5], the cluster location will be found based on the Hadoop configuration: HADOOP_CONF_DIR or YARN_CONF_DIR variables must be properly configured.

As an example, the following command corrects the dataset *sample.fastq* on a YARN cluster using the cluster deploy mode:

```
spark-submit --master yarn --deploy-mode cluster target/
    SparkEC.jar -in sample.fastq -out sample-output
```

3.4 Configuration

The SparkEC configuration can be tuned through a Java properties file. SparkEC provides a default properties file as a template (*config.properties.template*) at the *conf* directory. The available configuration parameters in this file are:

- `k` (integer). The k-mer length. The default value is 24.
- `seqLen` (integer). The average read length (used as hint). The default value is 0, which means that this value is auto-calculated.
- `numSeq` (integer). The number of input reads (used as hint). The default value is 0, which means that this value is auto-calculated.
- `splitMemoryConstant` (float). The `C` constant parameter used for the memory estimation of the split-based system. The default value is 5.25.
- `partitionSize` (integer). The partition size in MiB. The default value is 4.
- `sparkLogLevel` (string). Log level for the Spark runtime: FATAL, ERROR, WARN, INFO, DEBUG, TRACE. The default value is WARN.

[3] `https://spark.apache.org/docs/latest/cluster-overview.html`
[4] `https://spark.apache.org/docs/latest/spark-standalone.html`
[5] `https://spark.apache.org/docs/latest/running-on-yarn.html`

- `inputType` (string). The sequence format of the input dataset: FastQ, FastA, internal. The default value is FastQ.
- `shaveIgnore` (boolean). Whether or not to filter (ignore) reads that have 'N' bases from input. The default value is true.
- `numPinchCorrectAttempts` (integer). Number of iterations for the PinchCorrect phase. The default value is 1.
- `filter_P` (boolean). Whether the PinchCorrect filter of the LargeKmerFilter phase should be run or not. The default value is false.
- `filter_S` (boolean). Whether the SpreadCorrect filter of the LargeKmerFilter phase should be run or not. The default value is true.
- `numSpreadCorrectAttempts` (integer). Number of iterations for the SpreadCorrect phase. The default value is 1.
- `arm` (integer). The arm size to use for the SpreadCorrect phase. High values lead to high memory usage and best quality results. Use -1 as a flag to use the highest possible value. The default value is -1.
- `height` (integer). The height to use for the SpreadCorrect phase. The default value is 0.
- `scheme` (string). The arm scheme to run for the SpreadCorrect phase: CLA, ENV, GNV. The default value is null.
- `stackMax` (integer). The maximum stack to use for the SpreadCorrect phase (-1 to disable). The default value is -1.
- `stackMin` (integer). The minimum stack to use for the SpreadCorrect phase (-1 to disable). The default value is -1.
- `mergeIgnore` (boolean). Whether or not to merge ignored reads from input. The default value is false.
- `outputPreprocess` (boolean). Whether or not to output the PreProcess phase as a temporary result. The default value is false.
- `outputPinchCorrect` (boolean). Whether or not to output the PinchCorrect phase as a temporary result. The default value is false.
- `outputLargeKmerFilter` (boolean). Whether or not to output the LargeKmerFilter phase as a temporary result. The default value is false.
- `outputSpreadCorrect` (boolean). Whether or not to output the SpreadCorrect phase as a temporary result. The default value is false.
- `outputUniqueKmerFilter` (boolean). Whether or not to output the UniqueKmerFilter phase as a temporary result. The default value is false.
- `enablePinchCorrect` (boolean). Whether or not to enable the PinchCorrect phase. The default value is true.
- `enableLargeKmerFilter` (boolean). Whether or not to enable the LargeKmerFilter phase. The default value is true.
- `enableSpreadCorrect` (boolean). Whether or not to enable the SpreadCorrect phase. The default value is true.
- `enableUniqueKmerFilter` (boolean). Whether or not to enable the UniqueKmerFilter phase. The default value is true.

Finally, it may also be interesting to tune the Spark configuration in order to get the best performance. For instance:

- `spark.hadoop.validateOutputSpecs`: this option must be set to false if the output of individual phases is enabled.
- `spark.serializer`: it is highly recommended to set this option to use the Kryo serializer in order to take advantage of its features.

3.5 Compilation

In case you need to recompile the SparkEC source code, the prerequisites are:

1. Java Development Kit (JDK) version 1.8 (or above).
2. Apache Maven [19] version 3.1 (or above).
3. Hadoop Sequence Parser (HSP) library [20].

In order to build the executable JAR file, simply execute the following Maven command from within the SparkEC root directory:

```
mvn package
```

After a successful compilation, the resulting JAR file (*SparkEC.jar*) will be generated at the *target* directory. Note that the first time you execute the previous command, Maven will download all the plugins and related dependencies it needs to fulfill the command. From a clean Maven installation, this can take quite a while. If you execute the command once again, Maven will now have all what it needs, so it will be able to execute the command much more quickly.

4 SparkEC design

On the one hand, Figure S3 shows the UML class diagram of the overall SparkEC design, which has been simplified for clarity purposes (the classes for the pipeline phases are not included in the figure). On the other hand, Figures S4 and S5 show the UML class diagrams for the Node class and the IDNASequence interface, respectively. These new classes have been specifically introduced in SparkEC to optimize the encoding of DNA reads.

References

1. Goodwin, S., McPherson, J.D., McCombie, W.R.: Coming of age: Ten years of next-generation sequencing technologies. Nature Reviews Genetics **17**(6), 333–351 (2016)
2. Di Resta, C., Galbiati, S., Carrera, P., Ferrari, M.: Next-generation sequencing approach for the diagnosis of human diseases: Open challenges and new opportunities. eJIFCC **29**(1), 4–14 (2018)
3. Heather, J.M., Chain, B.: The sequence of sequencers: The history of sequencing DNA. Genomics **107**(1), 1–8 (2016)
4. McCombie, W.R., McPherson, J.D., Mardis, E.R.: Next-generation sequencing technologies. Cold Spring Harbor Perspectives in Medicine **9**(11), 036798 (2019)

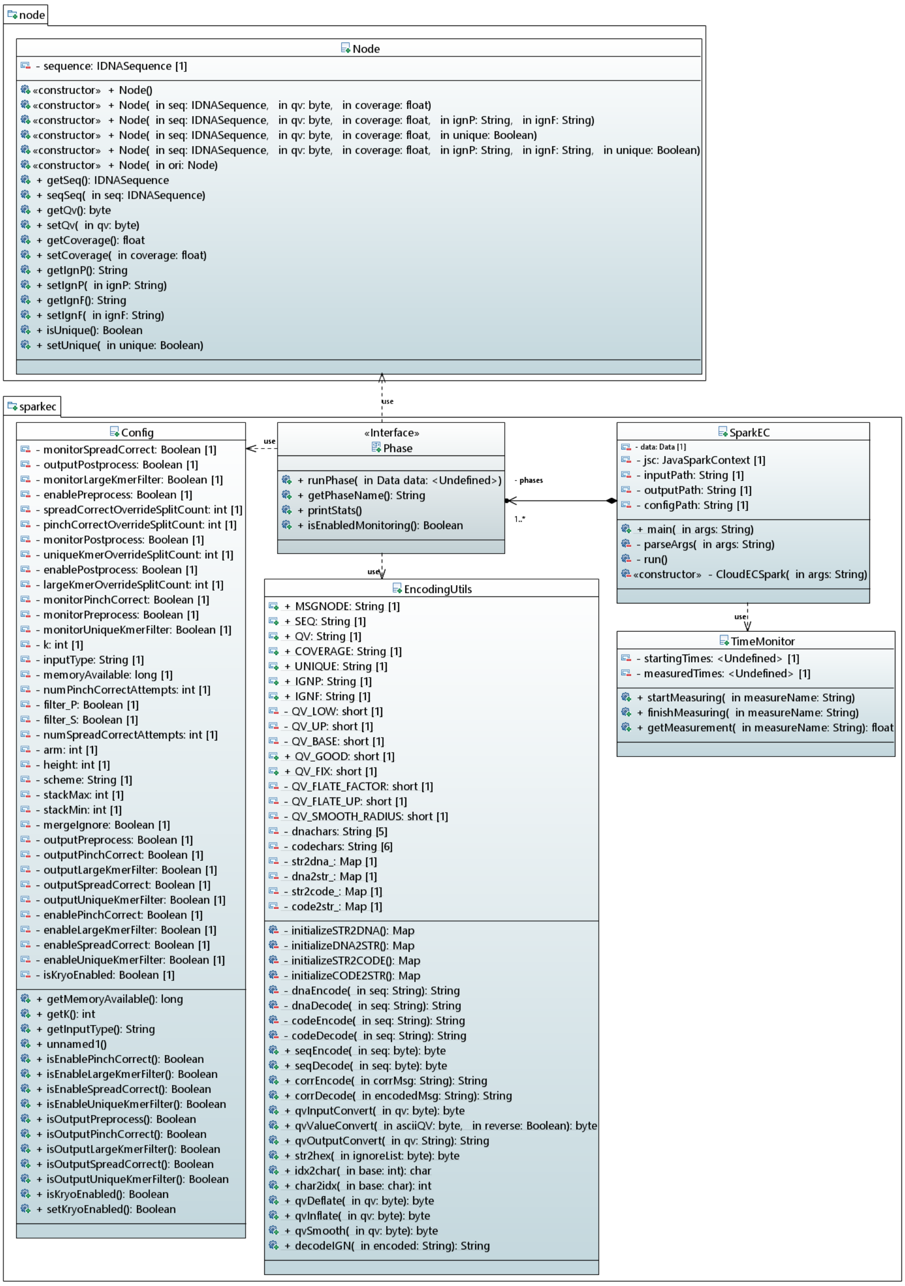

Figure S3. UML class diagram of SparkEC

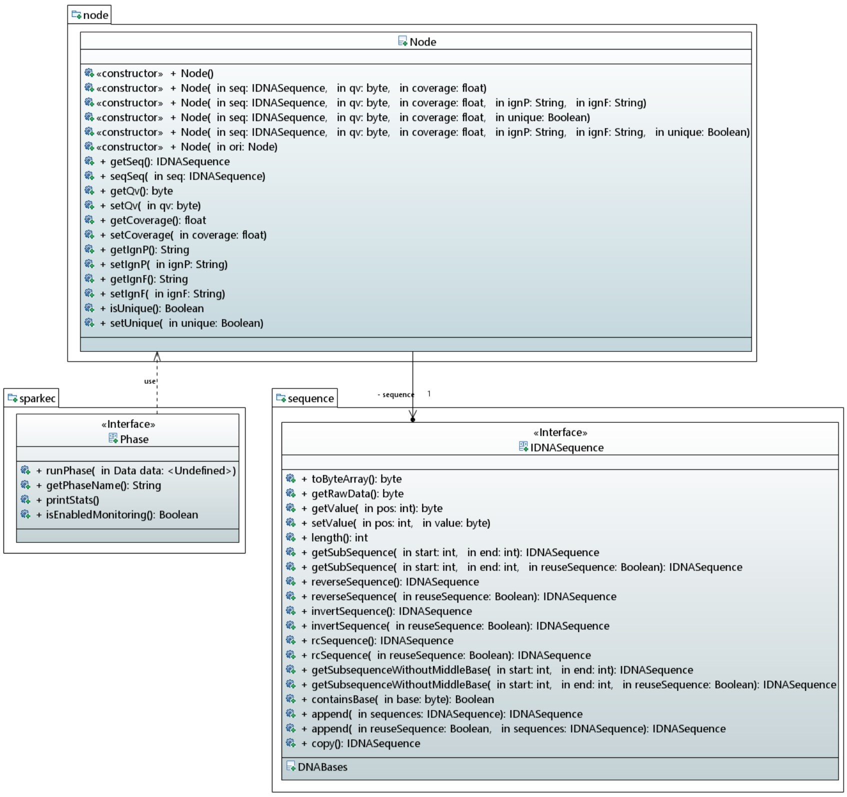

Figure S4. UML class diagram of Node class

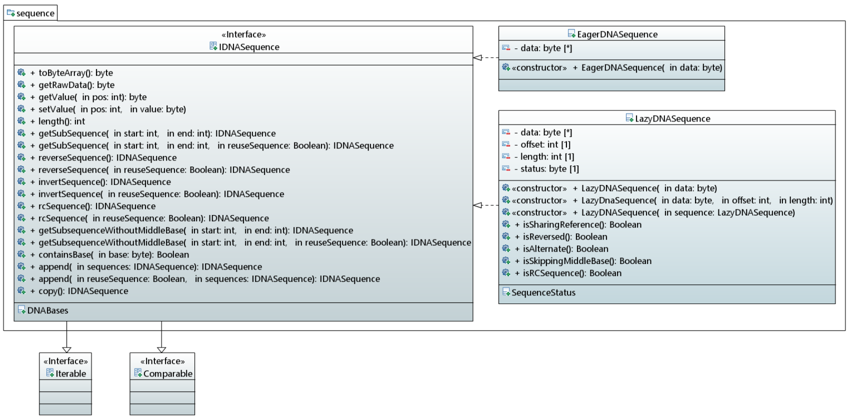

Figure S5. UML class diagram of IDNASequence interface

5. Shao, Z., Zhao, H., Zhao, H.: DNA assembler, an in vivo genetic method for rapid construction of biochemical pathways. Nucleic Acids Research **37**(2), 16 (2009)
6. Hadadian Nejad Yousefi, M., Goudarzi, M., Motahari, S.A.: IMOS: Improved Meta-aligner and Minimap2 on Spark. BMC Bioinformatics **20**(1), 51 (2019)
7. Glenn, T.C.: Field guide to next-generation DNA sequencers. Molecular Ecology Resources **11**(5), 759–769 (2011)
8. Yang, X., Chockalingam, S.P., Aluru, S.: A survey of error-correction methods for next-generation sequencing. Briefings in Bioinformatics **14**(1), 56–66 (2013)
9. Edgar, R.C., Batzoglou, S.: Multiple sequence alignment. Current Opinion in Structural Biology **16**(3), 368–373 (2006)
10. Dean, J., Ghemawat, S.: MapReduce: Simplified data processing on large clusters. Communications of the ACM **51**(1), 107–113 (2008)
11. The Apache Software Foundation: Apache Hadoop. [Online]. `https://hadoop.apache.org`. (accessed 15 September 2022)
12. Shvachko, K., Kuang, H., Radia, S., Chansler, R.: The Hadoop Distributed File System. In: Proceedings IEEE 26th Symposium on Mass Storage Systems and Technologies (MSST'10), pp. 1–10 (2010). Incline Village, NV, USA
13. Vavilapalli, V.K., Murthy, A.C., Douglas, C., Agarwal, S., Konar, M., Evans, R., *et al.*: Apache Hadoop YARN: Yet Another Resource Negotiator. In: Proceedings 4th Annual Symposium on Cloud Computing (SCC'13), pp. 1–16 (2013). Santa Clara, CA, USA
14. Ghemawat, S., Gobioff, H., Leung, S.-T.: The Google file system. SIGOPS Operating Systems Review **37**(5), 29–43 (2003)
15. Zaharia, M., Xin, R.S., Wendell, P., Das, T., Armbrust, M., Dave, A., *et al.*: Apache Spark: A unified engine for Big Data processing. Communications of the ACM **59**(11), 56–65 (2016)
16. Zaharia, M., Chowdhury, M., Das, T., Dave, A., Ma, J., McCauly, M., *et al.*: Resilient Distributed Datasets: A fault-tolerant abstraction for in-memory cluster computing. In: Proceedings 9th USENIX Symposium on Networked Systems Design and Implementation (NSDI'12), pp. 15–28 (2012). San Jose, CA, USA
17. Chung, W., Ho, J., Lin, C., Lee, D.T.: CloudEC: A MapReduce-based algorithm for correcting errors in next-generation sequencing Big Data. In: Proceedings IEEE International Conference on Big Data (IEEE BigData 2017), pp. 2836–2842 (2017). Boston, MA, USA
18. The Apache Software Foundation: Apache Spark's cluster mode overview. [Online]. `https://spark.apache.org/docs/latest/cluster-overview.html`. (accessed 15 September 2022)
19. The Apache Software Foundation: Apache Maven. [Online]. `https://maven.apache.org`. (accessed 15 September 2022)
20. Expósito, R.R., Mosquera, L.L., González-Domínguez, J.: Hadoop Sequence Parser library. [Online]. `https://github.com/UDC-GAC/hsp`. (accessed 15 September 2022)
